# Tumor suppressor FOXO3 regulates ribonucleotide reductase subunit RRM2B and impacts on survival of cancer patients

**DOI:** 10.18632/oncotarget.2044

**Published:** 2014-05-31

**Authors:** Er-Chieh Cho, Mei-Ling Kuo, Xiyong Liu, Lixin Yang, Yi-Chen Hsieh, Jinghan Wang, Yawen Cheng, Yun Yen

**Affiliations:** ^1^ Department of Clinical Pharmacy, School of Pharmacy, College of Pharmacy, Taipei Medical University, Taipei, Taiwan; ^2^ Department of Molecular Pharmacology, Beckman Research Institute, City of Hope, Duarte, CA, USA; ^3^ PhD Program for Neural Regenerative Medicine, College of Medical Science and Technology, Taipei Medical University, Taipei, Taiwan; ^4^ Department of Hepatobiliary Surgery, Navy General Hospital of Chinese PLA, Beijing, P. R. China; ^5^ Graduate Institute of Cancer Biology and Drug Discovery, College of Medical Science and Technology, Taipei Medical University, Taipei, Taiwan

**Keywords:** tumor suppressor FOXO3, RRM2B, cancer, biomarker

## Abstract

The role of Ribonucleotide reductase (RR) subunits in different cancers has been intensively studied in our laboratory. RRM2B was identified as a p53-inducible RR subunit that involves in various critical cellular mechanisms such as cell cycle regulation, DNA repair and replication, and mitochondrial homeostasis, etc. However, little is known about the p53-independent regulation of RRM2B in cancer pathology. In this study, we discovered tumor suppressor FOXO3 as the novel regulator of RRM2B. FOXO3 directly bound to and transcriptionally activated the promoter of RRM2B, and induced the expression of RRM2B at RNA and protein levels. Moreover, Overexpression of RRM2B and/or FOXO3 inhibited the proliferation of cancer cells. The cancer tissue microarray data also demonstrated a strong correlation between the co-expression of FOXO3 plus RRM2B and increased disease survival and reduced recurrence or metastasis in lung cancer patients. Our results suggest a novel regulatory control of RRM2B function, and imply the importance of FOXO signaling pathway in DNA replication modulation. This study provides the first time evidence that RRM2B is transcriptionally and functionally regulated independent of p53 pathway by FOXO3, and it establishes that FOXO3 and RRM2B could be used as predictive biomarkers for cancer progression.

## INTRODUCTION

Human ribonucleotide reductase (RR) plays a vital role in DNA synthesis and repair by catalyzing the de novo conversion of ribonucleoside diphosphates to deoxyribonucleoside diphosphates [1, 2]. In previous studies, we have extensively investigated the functional and structural regulation of RR subunits including RRM1, RRM2, and RRM2B (also known as p53R2) [2-9]. RRM2B has been demonstrated to be a p53-inducible RR subunit that functions in cell cycle regulation, DNA repair, mitochondrial homeostasis, and the suppression of metastasis [3, 10-14]. Mutation of the RRM2B gene causes severe mitochondrial DNA (mtDNA) depletion, which indicates that RRM2B plays a crucial role in the supply of dNTPs used for mtDNA synthesis [15]. RRM2B is involved in p53-dependent cell cycle regulation under DNA-damage conditions, and RRM2B can be regulated by p53 and p73 [12, 13, 16]. RRM2B and RRM1 were reported to associate with ataxia telangiectasia-mutated (ATM) and RRM2B was phosphorylated by ATM under genotoxic stress, which diminished RRM2B turnover [5]. Furthermore, RRM2B can suppress metastasis and inhibit proliferation in certain cancer cells [10, 17, 18], and RRM2B is the only RR subunit that has been identified as being capable of counteracting the actions of reactive oxygen species (ROS), which makes it unique [4, 7, 19-21]. However, the RRM2B signaling pathway is complex because the expression of RRM2B has been reported to correlate with either enhanced survival or an increase in the progressive phenotype in tissues of patients with various cancers [3, 22, 23].

The p53 tumor-suppressor pathway is largely defective in numerous human tumors [24-27]. RRM2B was identified as a critical p53-inducible RR subunit that is involved in tumor suppression [12, 28], but the p53-independent functional regulation of RRM2B in cancer remains mostly unknown. Therefore, investigating the functions of RRM2B and the regulation of RRM2B in p53-deficient cancers is crucial.

Forkhead box O 3 (FOXO3) is one of the most comprehensively characterized members of the FOXO family of transcription factors [29-31]. Under stress conditions, FOXO3 transactivates genes that are involved in cell cycle regulation, apoptosis, DNA repair, and antioxidation [29, 32, 33], which correlates with the aforementioned roles of RRM2B. Conversely, the localization and activity of FOXO transcription factors can be regulated transcriptionally or through posttranslational modifications such as phosphorylation, and all FOXO family members contain a conserved DNA-binding domain that binds to the FOXO consensus binding sequence, the forkhead-response element (FHRE) TTGTTTAC, on the promoter of target genes [32, 34]. A loss of FOXO function was observed in numerous human cancers and the conditional deletion of FOXOs led to tumorigenesis, which demonstrated that FOXOs act as tumor suppressors [35]. In this study, we tested the hypothesis that FOXO3 functions as a novel regulator of RRM2B.

## RESULTS

### FOXO3 regulates RRM2B level in p53-deficient cancer cells

**Figure 1:**
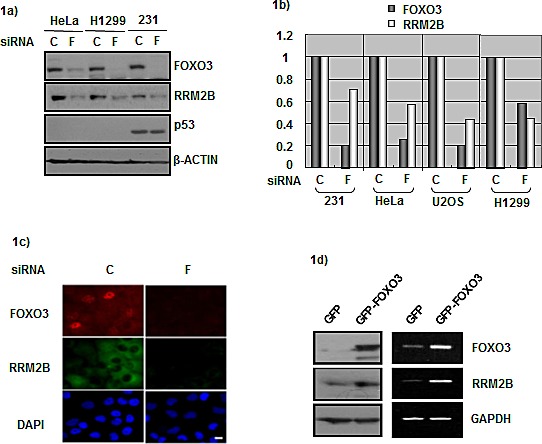
FOXO3 and RRM2B expression correlate in cancer cells a) HeLa, H1299, and MDA-231 cells were treated with either control siRNA (C) or FOXO3 siRNA (F) for 72 hours, and harvested for Western blot analysis with antibodies anti-FOXO3, anti-RRM2B, anti-p53, and anti-β-ACTIN (as loading control). b) Cells were treated with control or FOXO3 siRNA for 72 hours. Samples from U2OS, MDA-231 and HeLa cells were analyzed by qPCR. H1299 cells were analyzed by PCR, and then relative expression was analyzed by image J software. c) H1299 cells were seeded onto the coverslips in culture dishes. Cells were treated with siRNA for 72 hours, fixed and then immunoflourescence assay was performed. DAPI served as nuclear marker. d) H1299 cells were transfected with indicated plasmids, and then harvested for Western blots and RNA expression analysis 48 hours later.

We have sought to identify p53-independent regulators of RRM2B, and we hypothesized that FOXO3 regulates RRM2B. To test this, we introduced a FOXO3-targeting siRNA into cancer cells and examined whether downregulating FOXO3 affects RRM2B levels. To verify the p53-independent effect of FOXO3, we used p53-deficient cancer cells: HeLa (p53-degraded), H1299 (p53-null), and MDA-231 (p53-mutated) cells. RRM2B protein was downregulated in all 3 cell lines when FOXO3 expression was knocked down (Figure 1a). Next, to examine whether this correlation extended to the mRNA level, we used the 3 cancer cells and U2OS (wild-type p53) cells in the experiments. The cells were treated with the FOXO3 siRNA and the mRNA levels of the samples were measured. FOXO3 depletion led to RRM2B downregulation at both the protein and mRNA levels (Figures 1a and 1b), and this downregulation was also observed in immunofluorescence assays (Figure 1c). These results indicated that FOXO3 regulates RRM2B levels in cancer cells. Next, we tested whether the expression of excess FOXO3 produces the opposite effect, and we observed that FOXO3 overexpression led to an increase in RRM2B levels at both the RNA and protein levels in H1299 cells (Figure 1d). We obtained similar results using HeLa cells (data not shown).

### FOXO3 induces RRM2B promoter activity in a p53-independent manner

**Figure 2:**
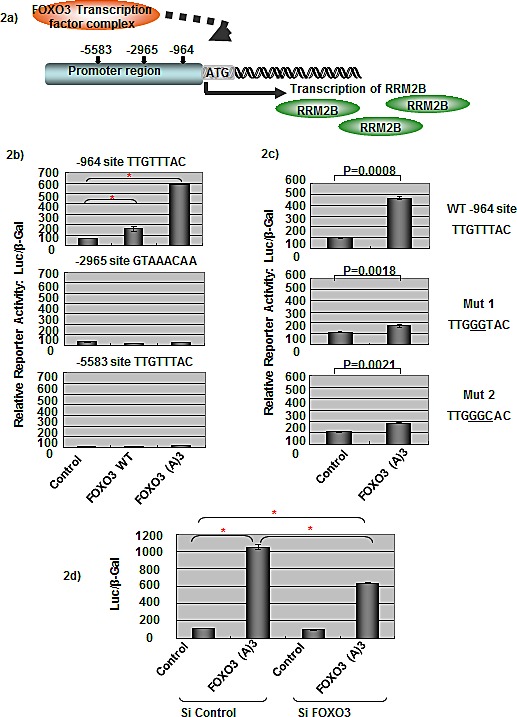
FOXO3 activates RRM2B transcriptional activity a) The graph shows three putative FHREs on the RRM2B promoter, where FOXO3 could potentially bind to and activate RRM2B transcription. b) Cells were co-transfected with different FHRE of RRM2B-Luc and either control, WT, or FOXO3(A)3 plasmids as indicated, and then reporter assays were carried out. (means ± SEM, *n* = 2). c) Cells were co-transfected with WT or RRM2B-Luc (-964) mutants and either control or FOXO3(A)3 plasmids as indicated, and reporter assays were performed. (means ± SEM, *n* = 2). d) Cells were co-transfected with WT RRM2B-Luc and either control or FOXO3(A)3 plasmids as well as treated with control or FOXO3 siRNA and analyzed by reporter assay 48 hours later. (means ± SEM, *n* = 3).

To understand how RRM2B is regulated under p53-deficient conditions further, we examined the promoter region of the RRM2B gene by using the programs TFSEARCH and PROMO. Several putative transcription factor-binding sites were identified within the 10-kb upstream region of the promoter. Among these sites, 3 putative FHREs (TTGTTTAC) were present upstream of the RRM2B ATG site; the presence of these sites, starting at −964, −2965, and −5583 bp, increased the possibility that the FOXO signaling pathway regulates RRM2B (Figure 2a).

We hypothesized that FOXO3 regulates RRM2B activity through the promoter of RRM2B, and tested this by performing luciferase-reporter assays in p53-negative H1299 cells. A control experiment demonstrated that FOXO3(A)3 (a constitutively active FOXO3 mutant, named (A)3) [36] activated pFHRE-Luc luciferase activity in our assay system (Supplementary Figure S1a). The RRM2B promoter constructs containing the 3 putative FHREs (−964, −2965, and −5583 sites; as described in the Materials and Methods section) were cotransfected into cells separately with either the control, wild-type FOXO3 (WT), or (A)3 plasmids, and the reporter activity was measured (Figure 2b). Both WT and (A)3 FOXO3 activated transcription activity of the RRM2B promoter containing the −964 site, and (A)3 exhibited higher activity than WT FOXO3 did (Figure 2b, upper panel). However, transcription was not activated for the other 2 putative FHREs (starting at −2965 and −5583) by either WT or (A)3 (Figure 2b). These results suggest that the −964 site is the functional FHRE present in the RRM2B promoter.

To confirm that the FHRE −964 site in the RRM2B promoter is specific for FOXO3-induced activation, we performed site-directed mutagenesis to mutate the reporter constructs; the mutated bases are underlined in Figure 2c. In the reporter assay, FOXO3-induced luciferase activity was drastically reduced in both mutant constructs, demonstrating that this FHRE present in the RRM2B promoter is a specific FOXO3-inducible site (Figure 2c).

We also confirmed that FOXO3 was responsible for inducing the reporter activity. When the FOXO3 siRNA was applied in the reporter assays, the luciferase activity induced by (A)3 was substantially reduced compared to the control level, which verified that the RRM2B promoter activity was specifically activated by FOXO3 (Figure 2d). In addition, we transfected HeLa and H1299 cells with the RRM2B-luciferase construct together with titrated amounts of FOXO3 plasmids, and the results further demonstrated the specificity exhibited by FOXO3 toward the RRM2B promoter (Supplementary Figures S1b and S1c).

Collectively, the results demonstrated that FOXO3 uses the FHRE in the RRM2B promoter and activates RRM2B transcription in a p53-independent manner.

### FOXO3 physically binds to the RRM2B promoter

**Figure 3:**
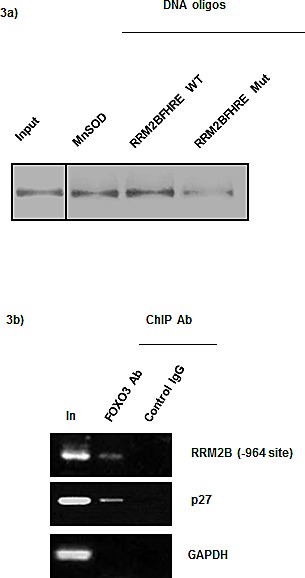
FOXO3 directly binds to the RRM2B promoter a) DNA pull-down assays were performed as described. Samples indicated were separated by SDS-PAGE and transferred onto PVDF membrane to analyze the [^35^S] intensity. Left lane 1 shows input control, FOXO3 protein. (*n* = 2) b) ChIP was performed with H1299 cell lysate and −964 primers in PCR. Control IgG served as negative control for IP. (*n* = 2) GAPDH served as negative control and p27 served as positive control.

Subsequently, we investigated whether FOXO3 directly binds to the promoter region of RRM2B by using *in vitro* DNA pull-down assays. Briefly, ^35^S-FOXO3 protein was synthesized and incubated with biotinylated double-stranded FHREs asindicated; DNA oligos derived from the RRM2B −964 site in the promoter region (RRM2B FHRE WT); DNA oligos containing a mutated RRM2B −964 site (RRM2B FHRE Mut); or, as a positive control, a recognized FHRE derived from the promoter of manganese superoxide dismutase (MnSOD). DNA-protein complexes were detected when the RRM2B and MnSOD oligos containing the wild-type FHRE site were used (Figure 3a), whereas the signals of these complexes were substantially reduced when FOXO3 was incubated with the mutant oligos. This result agrees with the data presented in Figure 2c and suggests that wild-type RRM2B −964 FHRE is specific and essential for FOXO3 binding *in vitro*.

To demonstrate that FOXO3 physically binds to the RRM2B promoter region containing FHRE in cells, we performed endogenous chromosome-immunoprecipitation (ChIP) assays by using H1299 cells, as described previously [37]. The results indicated that FOXO3 specifically bound to the promoters of RRM2B (the −964 site) and p27, but not to the promoters in the negative controls, which indicated that the interaction of FOXO3 with the promoter site is specific and independent of p53 (Figure 3b). Combined with the results shown in Figure 2, these data suggest that FOXO3 specifically binds to the −964 site in the RRM2B promoter and functionally activates RRM2B transcription. Furthermore, the results confirm that FOXO3 binds directly to the FHRE in the RRM2B promoter *in vitro* and in cells.

### The expression of FOXO3 and RRM2B inhibits the proliferation of cancer cells

**Figure 4:**
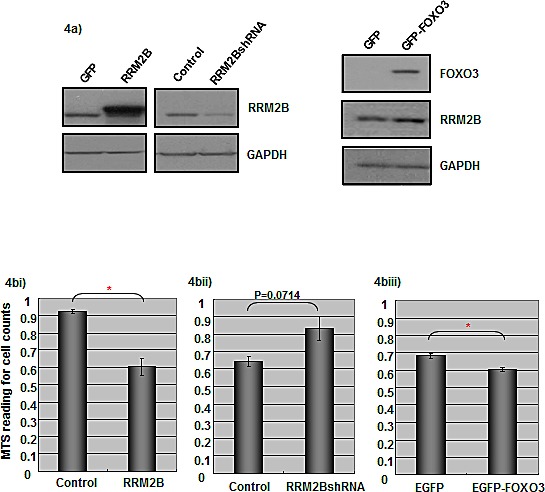
Expression of RRM2B and FOXO3 impacts on cancer cells proliferation a) Three pairs of H1299 cell lines expressing control vectors, RRM2BshRNA, RRM2B expressing vector, EGFP, and EGFP-FOXO3, were established and harvested for Western blots analysis with indicated antibodies. (*n* > 3) bi)-biii) Stable H1299 cells mentioned above as three pairs were seeded onto 96 well plates, and the proliferation of the cells was analyzed by MTS assay 72h later. 4bi), *n* = 3; 4bii) and 4biii), *n* = 2 (means ± SEM). Cells were seeded onto the 96 well plates and the proliferation of cells was analyzed by MTS assay 72h later. (means ± SEM, *n* = 2)

To investigate the consequence of FOXO3-mediated RRM2B regulation, we established 3 pairs of H1299 stable cell lines that expressed a control vector or RRM2BshRNA, a GFP control or an RRM2B-expressing vector, and EGFP or EGFP-FOXO3. The expression levels of proteins in the cells were examined using Western blot analysis (Figure 4a). Next, colorimetric MTS cell proliferation assays were performed to examine how RRM2B or FOXO3 expression affected the growth of cancer cells. Whereas RRM2B overexpression led to a reduction in the cell number, the expression of RRM2BshRNA led to an increase in MTS readings, compared with that of the respective controls; these results suggest that RRM2B inhibits the growth of cancer cells (Figures 4bi and 4bii). In accordance with these results, we observed that when FOXO3 was overexpressed, RRM2B was induced (Figure 1d and 4a) and the proliferation of cancer cells was inhibited (Figure 4biii). The results suggest that the expression of RRM2B and FOXO3 inhibits the proliferation of cancer cells, and that this effect is independent of p53. Finally, supporting these results, the p53-independent and RRM2B/FOXO3-mediated inhibition of cancer-cell growth was also demonstrated in colony formation assays, albeit to a lesser extent than in the MTS assay (Supplementary Figure S2).

### Coexpression of FOXO3 and RRM2B correlates with increased survival in lung cancer patients

**Figure 5:**
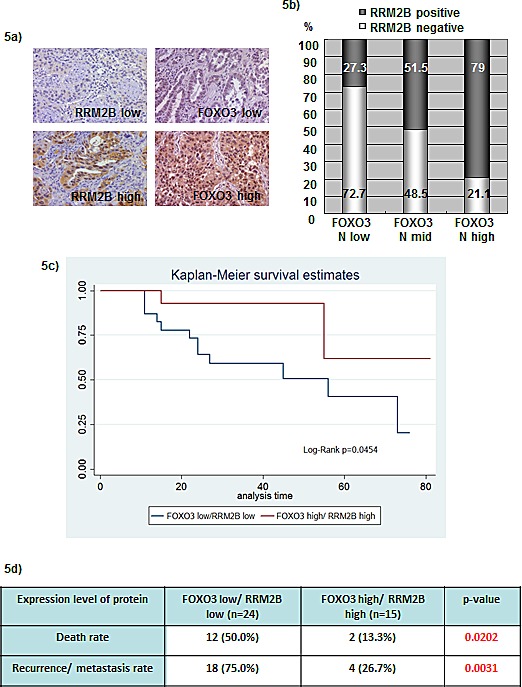
Coexpression of FOXO3 and RRM2B correlates with better disease survival in lung cancer patients a) Representative photographs from 63 lung cancer microarray samples showing examples for RRM2B low, RRM2B high, FOXO3 low, and FOXO3 high. IHC staining was carried out with anti-RRM2B (Rockland), and FOXO3 (Epitomics) antibodies. b) The expression correlation between FOXO3 and RRM2B was shown here and analyzed by Cochran-Armitage Trend test. The *P* < 0.0001 c) Patient samples were separated into two groups, FOXO3 high/RRM2B high and FOXO3 low/RRM2B low. Calculation and comparison of survival functions was analyzed by the Kaplan-Meier method. The differences in the time to event distribution were examined by the log-rank test. *P* = 0.0454 d) The death rate and recurrence/metastasis rate were analyzed between the two groups indicated, and the differences were significant. *P* = 0.0202 for the death rate and *P* = 0.0031 for the recurrence/metastasis rate.

To determine the consequence of RRM2B and FOXO3 expression in human cancers, immunohistochemistry (IHC) was performed using a lung-tissue microarray (n = 63) and the expression levels of RRM2B and nuclear FOXO3 were analyzed. Figure 5a presents examples of low and high expression of FOXO3 and RRM2B. We used the Cochran-Armitage Trend test to investigate the linear association between FOXO3 nuclear expression and RRM2B expression. The proportion of RRM2B-positive cells increased as the FOXO3 levels increased (*P* < 0.0001), and high expression of nuclear FOXO3 coincided with RRM2B expression in cancer patients (Figure 5b). The Kaplan-Meier method was used to compare disease survival between FOXO3 high/RRM2B high (high/high) and FOXO3 low/RRM2B low (low/low) groups, and the results indicated that the high/high group exhibited significantly higher survival compared with that of the low/low group in these cancer patients (*P* = 0.0454) (Figure 5c). Moreover, the high/high group exhibited a superior outcome compared with that exhibited by the low/low group in the analysis of the death rate and cancer recurrence or metastasis in these patients (Figure 5d).

Finally, we examined the difference between the demographics (sex and age) of the FOXO3/RRM2B low/low and high/high groups, and the results indicated no statistically significant differences (data not shown). The detailed calculation methods used are listed in Materials and Methods section.

Collectively, the results confirmed that FOXO3 is a novel regulator of RRM2B and revealed that FOXO3 can bind to the promoter region of the RRM2B gene and induce the transcription of RRM2B. Furthermore, FOXO3 and RRM2B inhibited the growth of cancer cells, and in accordance with these results, the IHC results obtained using tissues of human cancer patients indicated that coexpression of FOXO3 and RRM2B is correlated with increased survival and a reduced death rate and cancer recurrence or metastasis in lung-cancer patients.

## DISCUSSION

Since RRM2B was first identified as a p53 target, we have been considerably interested in determining whether the presence of wild-type p53 affects the FOXO3-dependent regulation of RRM2B. We examined the level of p53 expression in cancer patient samples used in Figure 5, and we determined FOXO3 and RRM2B expression levels in patient samples that exhibited strong p53 staining in IHC assays, which is often considered to indicate p53 mutation or deficiency. We obtained results indicating that patients expressing high/high FOXO3/RRM2B levels exhibited superior outcomes regarding the death rate and cancer recurrence or metastasis rate, compared with that of the patients in the low/low group (Supplementary Figures S3a and S3b). However, statistically significant data were not obtained from the weak-p53 group, which could be because of the low patient number (n = 15). Supporting the above results, the data in Figure 1b showed that FOXO3 regulates RRM2B levels in cancer cells despite the existence of wild-type p53. Furthermore, the reporter assay was applied in H1299 cells transfected with wild-type p53, and the results indicated that FOXO3 activates transcription of RRM2B promoter despite the present of p53 (Supplementary Figure S3c).

RRM2B was identified as a critical p53-inducible RR subunit that can be regulated by p53 and p73 [13]. However, the p53-independent regulation of RRM2B remains largely unknown. Intriguingly, RRM2B was expressed at basal levels in p53-null mouse embryonic fibroblasts (MEFs), although the p53-mediated induction of RRM2B expression in these cells was abolished [38]. We reported previously that RRM2B affects mitochondrial function similarly in p53-positive and -negative cancer cells, which suggests that in maintaining mitochondrial homeostasis, RRM2B does not depend on the presence of functional p53 [20]. Furthermore, RRM2B expression remains high in numerous types of p53-deficient cancer cells, which suggests that transcription factors other than p53 regulate the expression of RRM2B in these cells [17, 20]. In supporting the proposed hypothesis, this study has demonstrated for the first time that the tumor suppressor FOXO3 is a novel regulator of RRM2B that binds to the RRM2B promoter and activates the transcription of RRM2B in a p53-independent manner under physiological conditions.

Although several FOXO regulatory mechanisms have been identified, the serine/threonine kinase AKT is still considered to be a key regulator of the FOXO pathway [30]. Recently, the siRNA-mediated depletion of RRM2B was reported to result in the upregulation of AKT phosphorylation, which inactivates FOXO activity [39]. The current study established that FOXO3 activates RRM2B transcription by binding to the RRM2B promoter and positively regulating RRM2B levels in cells, which suggests that a feedback loop exists between the RRM2B and FOXO pathways.

FOXO3 and p53 share several common features. The subcellular localization and transcriptional activity of both proteins can be regulated by posttranslational modifications such as ubiquitination and phosphorylation [30, 40]. The 2 proteins share numerous downstream targets including the genes involved in stress resistance (the growth arrest and DNA damage-inducible 45, GADD45) and cell cycle control (p21). Recently, FOXO3 was identified as a direct target of p53 and was demonstrated to regulate the transactivation-independent proapoptotic activity of p53 [41-43]. The current results indicate that FOXO3 activates RRM2B transcription and expression in p53-deficient cells, as well as in cells expressing functional p53 (U2OS cells, Figure 1b; Supplementary Figure S3c). The RRM2B-mediated crosstalk between these 2 vital transcription factors in tumorigenesis and DNA repair regulation is fascinating and warrants further investigation.

As a key RR subunit, RRM2B plays a vital role in DNA synthesis and repair [1, 2, 15,38]. It was previously demonstrated that RRM2B is a p53-inducible RR subunit that plays a role in cell cycle regulation, DNA repair, mitochondrial homeostasis, and the suppression of metastasis [3, 10-13]. In accordance with these findings, the current results indicate that FOXO3 can activate and regulate RRM2B and inhibit the growth of cancer cells. Moreover, we obtained translational IHC data by using a tissue microarray prepared from samples of lung-cancer patients, and the results indicated that the expression of FOXO3 and RRM2B can be used as a predictive biomarker in future cancer diagnostics.

## MATERIALS AND METHODS

### Cell Lines and stable cell line production

Cell lines (ATCC) were cultured in DMEM medium containing 10% fetal bovine serum (Omega Scientific Inc.) and penicillin and streptomycin. All cells were incubated at 37°Cwith 5% CO2. Virus generation was described [19]. Stable cell lines were established by infection and then selection by either GFP marker (FACS sorting) 400 ug/ml G418, or 1.25 ug/ml puromycin selection for 5-10 days [19].

### Plasmids

pECE-FOXO3 (A)3 plasmid, EGFP, and EGFP-FOXO3 were kind gifts from Dr. Kato, Dr. Rama [36] and Dr. Marten Smidt. The MSCV-IRES-PURO-MCS vector for wild-type p53 was from Dr. Martine Roussel. For constructing MSCV-IRES-GFP-p53R2 plasmid, RRM2B-myc was excised from the pCMV-tag 1 vector [5], and the fragment was inserted into MSCV-IRES-GFP(MCS) vector with BamHI and SnaBI sites. RRM2BshRNA (shRRM2B-C) was described [19]. To clone the RRM2B reporter constructs, the RRM2B promoter region fragments contain three putative conserve FHREs, namely −964 (−1063~−753 bp ATG), −2965 (−3317~−2909 bp ATG), −5583 (−5614~−5302 bp ATG), were obtained by PCR and constructed separated into the pGL3 luciferase vector. Site-directed mutagenesis (Statagene) was carried out to mutate the FHRE (TTGTTTAC) on the −964 reporter construct, and the mutants contain TTGGGTAC (Mut1) and TTGGGCAC (Mut2) were sequenced. Primers are listed in the supplementary table 1.

### MTS assay

Cells were seeded onto the 96 well plates as 1500-2000 cells/well and then grow in the incubator for 72 hours before analysis. The number of the viable cells in proliferation was analyzed according to the manufacturer's instructions (Promega).

### Colony formation assay

Cells were seeded onto the 6 well plates as 100 cells/well, and then the cells were incubated for 10 days with medium changed every 3-4 days. When harvested, cells were rinsed with 1xPBS and then fixed and stained with 0.5% crystal violet in methanol: acetic acid (3:1) for 5 mins. Then, plates were rinsed and dried. Colonies were scored and analyzed for relative cell proliferation. In the bar charts, each “*n*” represents an independent experiment done in duplicate or triplicate. Please find other statistical information in the “Statistical methods” section.

### Plasmid and siRNA transfection

Lipofectamine 2000 or RNAiMAX (Invitrogen) were used for plasmid or siRNA transfection. Cells were harvested 48 hours after transfection of plasmids. siRNA treatment of cells was carried out using siRNA non-targeting control (sc-37007, Santa cruz), or FOXO3 siRNA (Santa Cruz) for 72 hours at 10-30 nM. siRNA sequences are listed in supplementary table 1.

### Luciferase Reporter assay

Reporter assay was performed as described [44]. Cells were transfected with expression vectors encoding control or FOXO3 proteins with RRM2B luciferase reporters together with β-gal to monitor transfection efficiency. Cells were harvested, lysed 48 h post-transfection and luciferase activity was measured (Promega). In the bar charts, each “*n*” represents an independent experiment done in duplicate or triplicate. Please find other statistical information in the “Statistical methods” section.

### Chromatin immunoprecipitation

Endogenous chromatin immunoprecipitation (ChIP) assay was performed with H1299 cells and anti-FOXO3 antibody (Santa Cruz) or IgG control antibody according to EZ-ChIP manufacturer's instructions (Millipore) [37]. PCR was performed with primers against GAPDH, p27 and three RRM2B promoter regions. Input DNA used was purified from the pre-cleared chromatin sample. Primers are listed in the supplementary table 1.

### Immunofluorescence

Cells were seeded on coverslips, fixed with 3.7% formaldehyde and permeabilized with 0.5% Tween20, stained with primary anti-FOXO3 (Santa Cruz), anti-RRM2B (Rockland), γ-H2AX (Active Motif) and secondary antibodies, washed, stained with DAPI as nuclear control, and then mounted onto the slides as described [45]. The slides were analyzed by Immunofluorescence microscope system. Scale bars indicate 10μM.

### DNA pull-down assay

Details of the DNA pull-down assay was performed as described [34]. Sequences of the oligonucleotides used are listed in the supplementary table 1. FOXO3a protein for DNA pull-down assay was transcribed and translated in TNT reticulocyte lysates (Promega) and was labeled with ^35^S-MET.

### Western blot analysis

Cell extracts were prepared with TNN buffer and analyzed as described [45]. Antibodies used were: RRM2B (produced by Rockland), anti-FOXO3a (Santa Cruz and Epitomics), γ-H2AX (Active Motif), p53 (Santa Cruz), GAPDH (Santa Cruz), VDAC1 (GeneTex), COX4 (GeneTex), and β-ACTIN (Sigma). Quantitative expression of γ-H2AX relative to GAPDH was analyzed by image J software and shown by numbers and a graph where applicable.

### Real-time PCR analysis

Total RNAs were isolated by using Trizol following the manufacturer's protocol. Then, RNA was reverse transcribed (Quanta) to obtain cDNA for PCR. cDNA was subjected to real-time PCR using the SYBR Green PCR reagents kit (Stratagene). Experiments were performed in triplicate for each data point. GAPDH was used as a control for normalization. Primers are listed in the supplementary table 1.

### Statistical methods

Student T-test was used for P value calculation. * stands for P < 0.05 if not specified. In all the bar charts, each “n” represents an independent experiment done in duplicate or triplicate for data with standard deviation, and the representative results are shown in this article. For the tissue microarray samples (n = 63), in order to assess if there is any difference between the demographics of the Low/Low group and the High/High group, Chi-Square test was conducted for the categorical variables while independent sample T-Test was utilized for the continuous variables. Calculation and comparison of survival functions was conducted using Kaplan-Meier method. The differences in the time to event distribution between the interested groups were examined using the log-rank test.

## SUPPLEMENTARY FIGURES AND TABLES




