# Supplementary material for: Tumor suppressor FOXO3 regulates ribonucleotide reductase subunit RRM2B and impacts on survival of cancer patients
Source: Oncotarget. 2014 May 31;5(13):4834–44. doi: 10.18632/oncotarget.2044 (PMC4148103; doi:10.18632/oncotarget.2044)
Supplement: Supplementary file 1 [file oncotarget-05-4834-s001.pdf]

Tumor suppressor FOXO3 regulates ribonucleotide reductase subunit RRM2B and impacts on survival of cancer patients

Supplementary Material

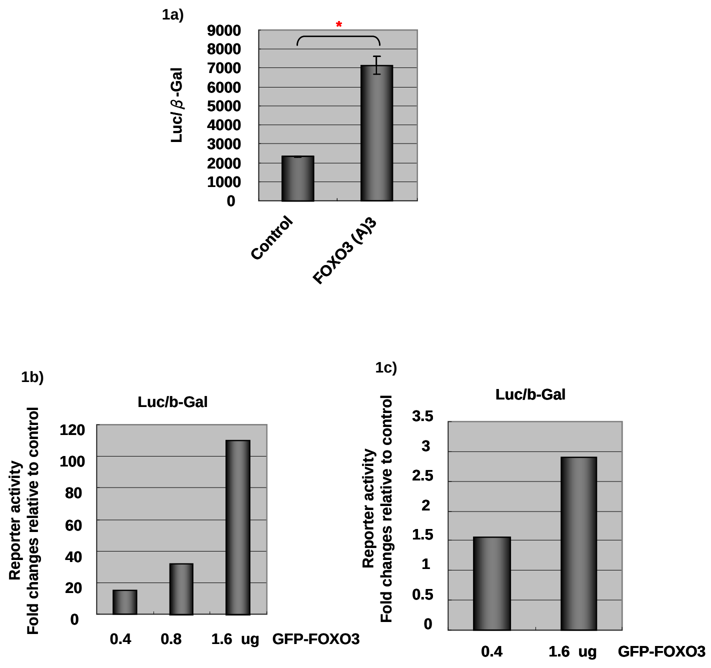

Supplementary S1: a) Luciferase reporter control experiment was firstly carried out by co-transfecting empty vector control or FOXO3(A)3 plasmid with pFHRE-Luc into the p53-negative H1299 cells. FOXO3(A)3 (named (A)3) expresses mutated FOXO3 protein which is constitutively active, and pFHRE-Luc contains repeated FOXO site sequences on the promoter region of the luciferase gene (30). HeLa (b)) and H1299 (c)) cells were co-transfected with RRM2B-Luc and either GFP control or GFP-FOXO3 plasmids for 48 hours, then the cells were harvested for reporter analysis. Fold changes relative to GFP control is shown. (n = 2) β-Gal was transfected in each assay to normalize the transfection efficiency in the reporter assays.

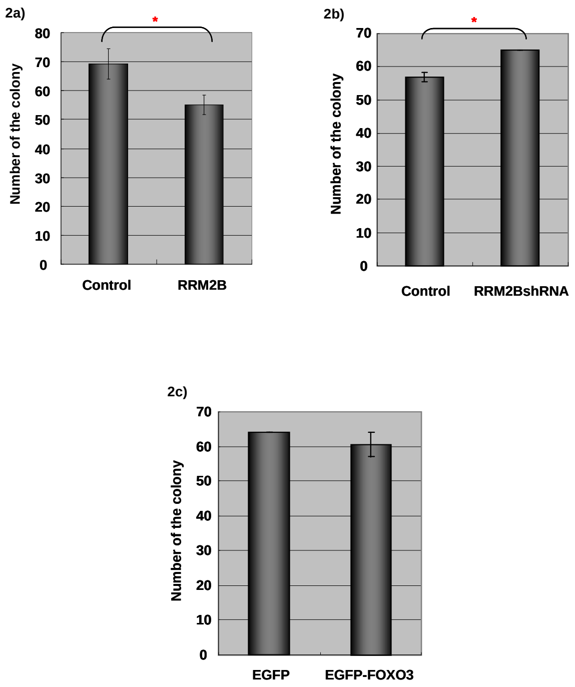

Supplementary S2: Colony formation assays were carried out with H1299 stable cells expressing control vector, RRM2BshRNA, GFP, RRM2B expressing vector, EGFP, and EGFP-FOXO3 as indicated. a), *n* = 4; b), *n* = 2; c), *n* = 3.

3a)

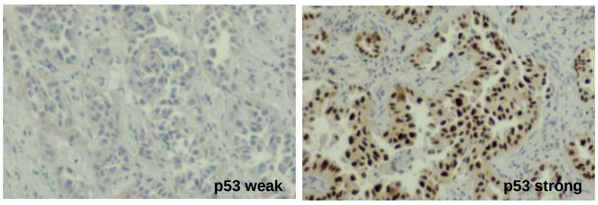

3b)

Expression level of protein	FOXO3 low/ RRM2B low (n=14)	FOXO3 high/ RRM2B high (n=10)	p-value
Death rate	7 (50%)	1 (10%)	0.0297
Recurrence/ metastasis rate	11 (78.6%)	2 (20.0%)	0.0054

3c)

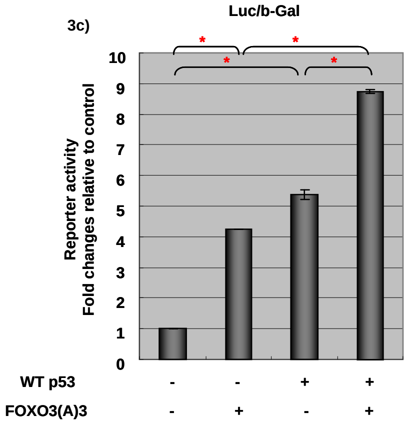

Supplementary S3: a) Representative photographs from lung cancer microarray samples showing examples for p53 weak and p53 strong IHC staining using p53 (DAKO) antibody. b) Samples with strong p53 staining were further analyzed by FOXO3 and RRM2B expression as indicated. The death rate and recurrence/metastasis rate were analyzed between the two groups, and the differences were significant. P = 0.0297 for the death rate and P = 0.0054 for the recurrence/metastasis rate. c) H1299 stable cell lines expressing control vector or WTp53 were used in the reporter assay. Cells were co-transfected with RRM2B-Luc and either control or FOXO3(A)3 plasmids as indicated. Then, the cells were harvested for reporter analysis 48 hours later. Fold changes of reporter activity relative to control is shown. (means±SEM, n=2) β-Gal was transfected in each assay to normalize the transfection efficiency in the reporter assays.
